# Human Keratinocyte Responses to Woodsmoke Chemicals

**DOI:** 10.1021/acs.chemrestox.3c00353

**Published:** 2024-04-10

**Authors:** Noreen Karim, Yatian Yang, Michelle Salemi, Brett S. Phinney, Blythe P. Durbin-Johnson, David M. Rocke, Robert H. Rice

**Affiliations:** † Department of Environmental Toxicology, University of California Davis, Davis, California 95616-8588, United States; ‡ Proteomics Core Facility, University of California Davis, Davis, California 95616, United States; § Division of Biostatistics, Department of Public Health Sciences, Clinical and Translational Science Center Biostatistics Core, University of California Davis, Davis, California 95616, United States

## Abstract

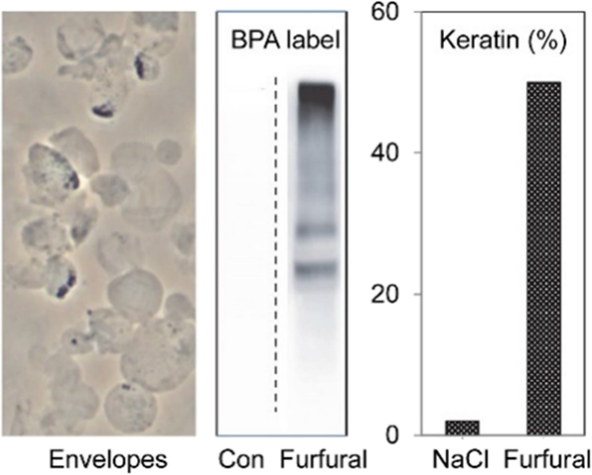

Air pollution consists of complex mixtures of chemicals with serious deleterious health effects from acute and chronic exposure. To help understand the mechanisms by which adverse effects occur, the present work examines the responses of cultured human epidermal keratinocytes to specific chemicals commonly found in woodsmoke. Our earlier findings with liquid smoke flavoring (aqueous extract of charred wood) revealed that such extracts stimulated the expression of genes associated with oxidative stress and proinflammatory response, activated the aryl hydrocarbon receptor, thereby inducing cytochrome P4501A1 activity, and induced cross-linked envelope formation, a lethal event ordinarily occurring during terminal differentiation. The present results showed that furfural produced transcriptional responses resembling those of liquid smoke, cyclohexanedione activated the aryl hydrocarbon receptor, and several chemicals induced envelope formation. Of these, syringol permeabilized the cells to the egress of lactate dehydrogenase at a concentration close to that yielding envelope formation, while furfural induced envelope formation without permeabilization detectable in this way. Furfural (but not syringol) stimulated the incorporation of amines into cell proteins in extracts in the absence of transglutaminase activity. Nevertheless, both chemicals substantially increased the amount of cellular protein incorporated into envelopes and greatly altered the envelope protein profile. Moreover, the proportion of keratin in the envelopes was dramatically increased. These findings are consistent with the chemically induced protein cross-linking in the cells. Elucidating mechanisms by which this phenomenon occurs may help understand how smoke chemicals interact with proteins to elicit cellular responses, interpret bioassays of complex pollutant mixtures, and suggest additional sensitive ways to monitor exposures.

## 1 Introduction

Recent analyses reveal that air pollution is a major killer in the human population, fourth in the ranking of death from all causes.^1^ Fine particulates (PM2.5) derived by combustion are responsible for increased mortality from lung and cardiopulmonary disease,^2^ where reduction of exposure lowers the risk.^3^ Among the combustion products in air pollution, smoke from biomass burning is a major source worldwide of volatile organics.^4^ Frequently encountered, it is of increasing concern, particularly in areas prone to wildfire,^5^ and merits further investigation of pathological targets, especially in susceptible populations.^6−8^ Woodsmoke aerosols have a complex mix of chemicals that have biological effects contributing to their toxicity.^9^ The connection between air pollutant exposure and deleterious health consequences is well established by epidemiology, but the specific pollutant chemicals and their mechanisms of action need further elucidation.

While studies of its deleterious effects have focused on lung function and cardiovascular disease, air pollution can affect other organ systems. For instance, it can exacerbate chronic inflammatory skin conditions^10−12^ and accelerate skin aging.^13^ Studying keratinocytes as targets likely will reveal responses that are relevant to this cell type in the upper respiratory tract, oral cavity, and trachea (appearing in regions subject to pollution exposure) and more generally to other target cell types. Airborne polycyclic aromatic hydrocarbons, insoluble in aqueous media, are well-known to be mutagenic and highly damaging^14^ but are not directly related to proinflammatory effects and lung toxicity.^15,16^ The water-soluble fraction of smoldering woodsmoke, used for food flavoring, for example, has the advantage of greatly reduced polycyclic aromatic hydrocarbon content and, thus, is anticipated to have lower health risk than traditional smoking procedures.^17^ It also has natural antimicrobial activity, an advantage for food preservation.^18^ However, it contains a variety of phenols, phenol ethers, catechols, carbonyls, furfural, and homologues with uncertain health effects.^19^ The aqueous fraction can be studied in a cell culture, where the exposed human epidermal keratinocytes exhibit deleterious effects. Noteworthy responses upon treatment include induction of cytochrome P4501A1, markers of oxidative stress and inflammatory mediators, and, at a higher concentration, cross-linked envelope formation, a lethal event.^20^

Although health effects correlate closely with the PM2.5 level, recent studies have demonstrated different levels of toxicity among different chemical classes as well as PM from different sources, suggesting potentially distinctive effects from PM with different chemical compositions. Profoundly different effects of pollutant chemicals on keratinocyte envelope formation have recently been observed.^20^ Ordinarily, a variety of proteins are enzymatically cross-linked by keratinocyte transglutaminase (TGM1) during terminal differentiation,^21^ forming a scaffold for the attachment of lipids responsible for the epidermal lipid barrier.^22^ Defective envelope formation due to insufficient TGM1-induced cross-linking, resulting in a defective barrier, is a major cause of the potentially debilitating skin disease autosomal recessive congenital ichthyosis.^23^ Our previous work indicates that the exposure of keratinocytes to the woodsmoke extract perturbs protein incorporation into envelopes and in this way could affect dermatological disorders.^20^

Since woodsmoke extract is a complex chemical mixture, the present work has focused on studying commercially available chemicals (phenols, catechols, and carbonyls) known to be or similar to those in aqueous smoke extract.^19^ The hypothesis being explored is that individual chemical moieties in the extract are responsible for the specific responses observed. Since various chemicals could overlap in eliciting each of the several biological responses we observed previously, we envisioned that the present strategy would complement and possibly more efficiently reveal classes of active chemicals than multifactorial assays of smoke fractions. The results of the present work indicate that cellular responses are explainable at least in part by the superposition of different chemicals eliciting different responses, where certain carbonyls appear especially versatile. Information on chemical interactions in cells can help rationalize modeling observations that individual chemical analyses may not capture the effects of chemical mixtures.^24^

## 2 Experimental Procedures

### 2.1 Chemicals

Biotin pentylamine (BPA, 95%) was obtained from Alpha Chemistry. 2,3,7,8-Tetrachlorodibenzo-p-dioxin (TCDD) was obtained from the NCI Chemical Carcinogen Repository. X537A was a generous gift from Hoffman-La Roche Chemical. 7-Ethoxyresorufin (95%), 3-methoxycatechol (99%), menadione (>98%), cyclohexanone (>99%), 1,3-cyclohexanedione (97%), and 1,4-cyclohexanedione (98%) were obtained from Sigma-Aldrich. Catechol (>99%), 2(5H)-furanone (>93%), camphor (>95%), and m-cresol (>98%) were purchased from TCI. Pyrogallol (98%) and maltol (97%) were obtained from AK Scientific. Syringol (98%) and guaiacol (98%) were from Ark Pharmaceuticals. Furfural (98%) was from J&K Scientific. 2-OH-3-Methylcyclopenten-2-en-1-one (95%) was from Enamine. Resorufin was from ICN Biomedicals. Since chemicals must be handled safely, smoke chemicals were manipulated in fume or culture hoods to avoid investigator exposure.

### 2.2 Cell Culture

Human epidermal keratinocytes (passage 3–8) (HEP) were cultured in Dulbecco-Vogt Eagle’s and Ham’s F-12 media (2:1 ratio) containing 5% fetal bovine serum, 10 ng/mL epidermal growth factor, 0.4 μg/mL hydrocortisone, 0.18 mM adenine, and 5 μg/mL each of insulin and transferrin.^25,26^ Human embryonic kidney (HEK 293) cells were cultured using a 2:1 mix of Dulbecco-Vogt Eagle’s and Ham’s F-12 media containing 10% fetal bovine serum and antibiotics (penicillin and streptomycin). The cells were grown until confluence before treatment with individual chemicals for envelope analysis or for harvesting cell extracts for other assays or analyses. To provide a contrast with the smoke chemicals, the cultures were treated overnight in some cases with 0.8 M NaCl or 0.17 mM ionophore X537A, which permeabilize the cells and activate transglutaminase cross-linking of envelopes.^27^

### 2.3 Chemical Induction of Envelopes

Cells were treated with smoke chemicals at different concentrations either by direct addition to the serum-free medium in which they were incubated for treatment or from a dilution in acetonitrile (e.g., syringol was 50 mg/mL, and furfural was 88 μL/mL in acetonitrile). In previous work,^9^ supplementation of the serum-free medium with up to 20% acetonitrile had no effect on the cell response. Cultures were then treated with 2% SDS–25 mM DTT (dithiothreitol)–50 mM Tris (pH 8) for 24 h at room temperature to obtain soluble and insoluble protein fractions. Envelopes, which resist dissolution by the detergent, were isolated by centrifugation and washed with 0.2% SDS three times before measuring their protein content using bicinchoninic acid.^28^ The values were adjusted for negative control samples (cells passaged parallel to the treatment group but not treated with chemicals). EC50 values were calculated from the dose–response curves as halfway between the adjusted baseline and maximal activity for each chemical.

### 2.4 Lactate Dehydrogenase Assay

Cultures treated with different concentrations of chemicals for 0 and 24 h were tested for the intactness of their cell membranes by measuring lactate dehydrogenase (LDH) activity in the medium with the Pierce LDH cytotoxicity assay kit using standard protocols (Thermo Fisher 88953). The assay was performed in triplicate for each sample. The samples and reaction/substrate were mixed in a 1:1 ratio and incubated at 37 °C for 30 min in the dark. Reactions were stopped by adding Stop Solution to each well, and the A490 and A650 nm values were measured.

### 2.5 Biotin Pentylamine Assay

Biotin pentylamine ((5-(biotinamido) pentylamine), BPA) incorporation assay was performed to detect the effect of the chemicals on the cross-linking of proteins in the presence and absence of transglutaminase-1 (TGM1) enzyme activity.^29^ Keratinocytes at confluence were rinsed with phosphate-buffered saline, harvested in 0.1 M HEPES buffer (pH 7.4), lysed by freeze–thawing, and homogenized by brief sonication, Typically, the homogenate from one 6 cm culture was distributed equally in seven aliquots of 100 μL for the assay. BPA (10 mM) along with the indicated smoke components was added, followed by incubation at 37 °C for 35–40 min. After incubation, SDS (2% w/v) and DTT (20 mM) were added, and the samples were incubated for 5 min in a boiling water bath. Samples were run on SDS-PAGE, blotted on an Immobilon transfer membrane (IPVH00010), followed by overnight incubation with horseradish peroxidase-linked antibiotin antibody (Cell Signaling Technology, 7075S). Chemiluminescent horseradish peroxidase substrate (Pierce ECL Western blotting substrate) was used to visualize the bound antibodies on a MyECL Imager (Thermo Fisher 62236X). To show amine incorporation into protein independent of cellular transglutaminase, cultures were treated with 20 mM iodoacetamide for 1 h before harvest to inactivate this activity.^20^

### 2.6 Sample Processing for Proteomic Profiling

The proteomic profiles of the envelopes (insoluble fraction) and solubilized fractions of the cells were compared. To 10 cm triplicate confluent cultures were added furfural (15 mg/mL), syringol (2 mg/mL), or NaCl (0.8 M) for 24 h. Afterward, media containing the treatment chemicals were removed, and the cells were rinsed with phosphate-buffered saline and then harvested by scraping and stored in 0.3 mL of 10 mM Tris -1 mM EDTA (TE) at −80 °C until use. Before processing them for digestion, the cell samples were suspended in 2 mL of 2% sodium dodecyl sulfate (SDS)–100 mM sodium phosphate buffer (pH 7.8)–50 mM DTT and heated at 95 °C for 5 min. The samples were stirred magnetically for 30 min and centrifuged at 18,000 *g* for 5 min, and the supernatants were collected. The pellets were resuspended in 2 mL of the SDS–phosphate–DTT buffer, and the above reduction process was repeated three more times, except that in the third and fourth reductions the volume was increased to 8 mL. A fifth reduction using a total volume of 1 mL was followed by the addition of iodoacetamide (100 mM) and stirring at room temperature for 45 min in the dark. Envelope samples were centrifuged, and the supernatants were discarded, followed by rinsing the pellets twice with 70% ethanol. The pellets were resuspended in 50 mM ammonium bicarbonate–10% acetonitrile solution, and 50 μg of reductively methylated trypsin was added.^30^ Simultaneously, 200 μL each of the first and second supernatants pooled together for the samples was alkylated with 100 mM iodoacetamide, followed by precipitation of the proteins by the addition of ethanol to 70%.^31^ The samples were stirred for 3 days with a fresh addition of trypsin each day. On the fourth day, the digested samples were centrifuged twice at 21,000 *g* for 30 min to remove particulates, and the supernatants were collected for mass spectrometric analysis. Trypsin treatment of the envelope and the corresponding SDS-solubilized protein fractions yielded 92 ± 6% of the material in the digestion supernatant.

### 2.7 Proteomic Profiling, Label-Free Quantitation, and Statistical Analysis

The peptide digests were randomized and subjected to LC-MS/MS using a Thermo Scientific Q Exactive Plus Orbitrap mass spectrometer, as described previously.^32^ The raw proteomic data files were searched against a Human Uniprot Proteomic database (UP000005640) supplemented with decoy sequences, and the data were analyzed in Scaffold 5.0.1 as previously described.^21^ Exclusive spectral counts for the proteins were used to compare the proteomes of the groups. The Scaffold file and raw data are available at the MassIVE Proteomics repository (https://massive.ucsd.edu/) with MassIVE id number MSV000093227 and Proteome Exchange (http://www.proteomexchange.org/) with the data set identifier no. PXD046590.

Proteins with average expression across the samples less than one count were filtered out prior to statistical analysis. Differential protein expression analyses were conducted based on spectral counts^20,33−35^ using the limma–voom Bioconductor pipeline,^36^ which was originally developed for RNA sequencing data (limma version 3.46.0 and edgeR version 3.32.0). Normalization factors were calculated using TMM. The model used in limma included the effects for individual and batch. Analyses were conducted using R version 4.0.2 (2020-06-22). Data were read from Table S1. The program and results of the statistical testing are provided as Supporting Information. Label-free quantitation, based on the top three peptides for each protein, was performed using the Q-module function of PEAKS Studio, as previously described.^32^ Values were normalized to 100 for each sample. Statistical testing employed ANOVA with Tukey HSD using either Stata 9.2 (Figures 2, 6, 7, and S4) or Statistics Kingdom online calculator (Figure 5) https://www.statskingdom.com/180Anova1way.html.

### 2.8 Measurement of Envelope Protein Content

Treated for 1 day in 2 mL of serum-free medium with chemical concentrations just sufficient to induce maximal envelope formation, 6 cm cultures were harvested with the addition of PBS to 5 mL, and the cells were recovered by low-speed centrifugation. The pellets were rinsed twice with 5 mL of PBS, disrupted by sonication to a fine suspension, adjusted to 2% in SDS, and sonicated again. An aliquot (0.1 mL) was removed for protein measurement, and the remaining cell material was brought to 50 mM each in DTT and sodium phosphate (pH 7.8). After end–overend mixing overnight, the pellet was recovered by centrifugation (10,000 *g* for 4 min) and rinsed six times with 0.1% SDS. (The final rinse had a negligible protein content.) The pellet was resuspended in 1.25 mL of 0.1% SDS for protein assay using bicinchoninic acid.^28^

### 2.9 Real-Time PCR (qPCR)

Cultures were harvested in TRIzol, and the total cellular RNA was isolated. cDNA was synthesized from the RNA extract using a high-capacity cDNA reverse transcription kit (Applied Biosystems, Cat. 4368814). cDNA was used with qPCR-based TaqMan gene expression assays for CXCL8, CYP1A1, CYP1B1, GCLM, HMOX1, NQO1, PTGS2(COX2), and TXNRD along with GAPDH and/or GUSB as housekeeping genes for normalization. qPCR runs were performed on a BioRad CFX96 C1000 Touch Thermal Cycler. Under optimal treatment conditions, furfural was maximally inducing transcriptionally when the cells were exposed at 2 mg/mL for 24 h.

### 2.10 Aryl Hydrocarbon Receptor (AHR) Activation

Recombinant human and rat hepatoma cells (HG2L6.1c1 and H4L1.1c4) were grown in minimal essential medium α containing 10% fetal bovine serum. These cells contain a stably transfected AhR-responsive firefly luciferase reporter gene plasmid (pGud-Luc6.1 or pGudLuc1.1).^37^ After 24 h incubation with various chemicals at indicated concentrations at 37 °C in 96-well plates, cells were rinsed twice with phosphate-buffered saline and lysed with Promega passive lysis buffer. Luciferase activity was measured using an Orin microplate luminometer (Berthold Technologies, Bad Wildbad, Germany) with an automatic injection of Promega-stabilized luciferase reagent as previously described.^38^ Luciferase activity was corrected for background activity from DMSO-treated cells and expressed as a percent of the luciferase activity obtained with a maximally inducing concentration (10 nM) of TCDD.

### 2.11 Ethoxyresorufin O–Deethylase Assay

Induction of CYP1A1 was measured on the enzymatic level using ethoxyresorufin-O-deethylase (EROD) assay, a measure of the CYP1A-mediated O-deethylation of 7-ethoxyresorufin to form the fluorescent product resorufin; hence, measurement of the fluorescence is a direct measure of the enzyme activity.^39,40^ Cells were treated with individual smoke components for 18–24 h, after which 7-ethoxyresorufin (4 mM) was added to the culture medium. The cells were incubated for another 2 h, and the medium was harvested. Fluorescence (excitation at 560 nm and emission at 600 nm) was measured with a SpectraMax iD3 multimode microplate reader (Molecular Devices, San Jose, CA USA). Since concentrations approaching EC50 for envelope formation are toxic and can reduce transcription,^29^ cells were treated with concentrations 10-fold lower than their respective EC50 values for envelope formation.

## 3 Results

### 3.1 Envelope Formation: Role of Permeabilization

**Figure 1:**
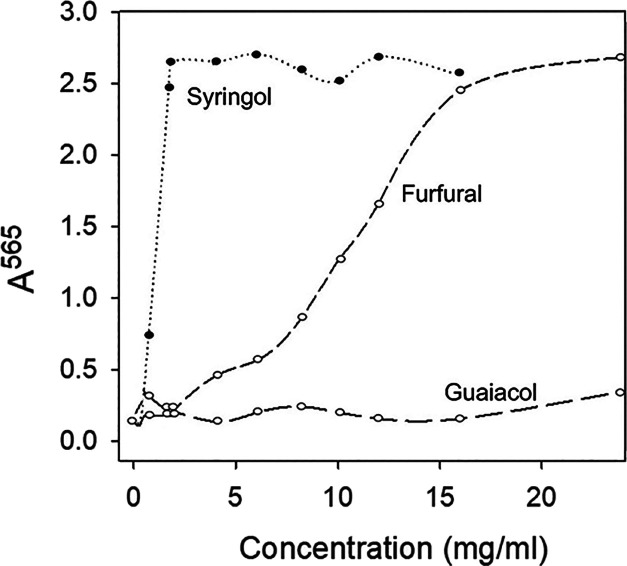
Concentration dependence of envelope formation for the three representative constituent smoke chemicals. Cultures in 24-well plates were treated for 1 day at 37 °C with guaiacol, syringol, or furfural at the indicated concentrations in serum-free medium, and then nonenvelope proteins were dissolved by the addition of SDS to 2% and DTT to 20 mM for an hour or more. The envelope structures were rinsed several times in 0.1% SDS, and protein levels were quantitated.

**Figure 2:**
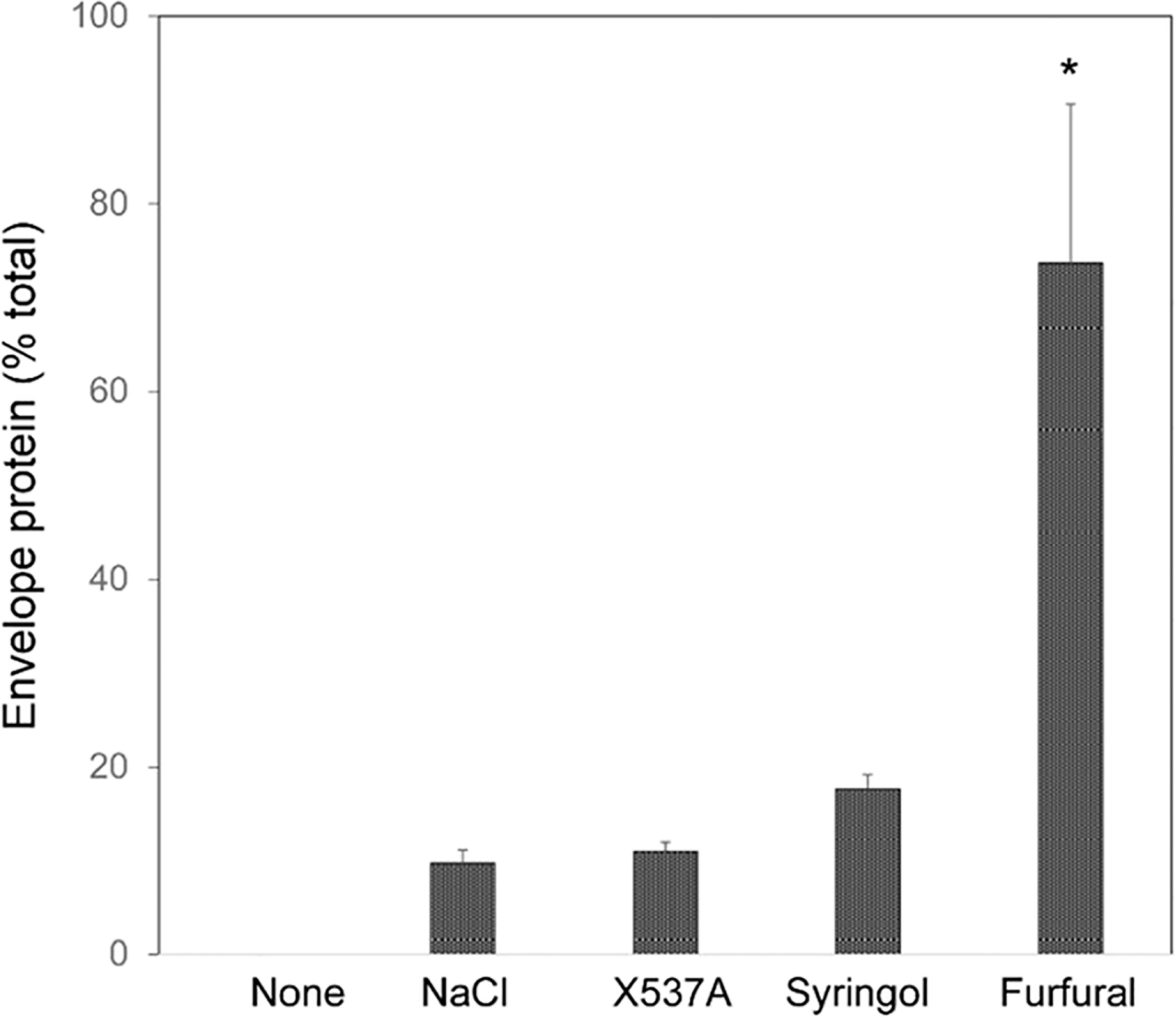
Percentage of total cell protein incorporated into SDS/DTT-insoluble envelopes. Cultures were incubated in 2 mL of serum-free medium for a day with the addition of NaCl (93 mg), ionophore X537A (200 μg), syringol (5 mg), furfural (17 mg), or no addition (none). Total and SDS-/DTT-insoluble protein fractions were quantitated in triplicate cultures. The values for furfural were significantly higher than the others (* *p* < 0.001).

An initial survey showed that chemical components of woodsmoke differed dramatically in the ability to induce protein envelopes in the keratinocyte cultures during exposure for 1 day. As shown in Figure 1, syringol induced envelope formation with an EC50 of about 1 mg/mL in culture medium, while furfural was an order of magnitude lower in potency and guaiacol appeared ineffective. A survey of other smoke chemicals also revealed a wide range of potencies (Table 1). Several compounds in addition to guaiacol were inactive, and several others were as potent as or more potent than syringol. Moreover, the degree of protein incorporation into SDS/DTT-insoluble envelope structures also varied with the treatment (Figure 2). Untreated epidermal cell cultures had little if any such material, while those treated with ionophore X537A or 0.8 M NaCl incorporated ≈10% of cellular protein into envelopes. By contrast, cells treated with furfural and syringol at concentrations just sufficient to give maximal envelope formation (Figure 1) had ≈70% and nearly 20%, respectively, of their total protein incorporated into envelopes, a difference of nearly threefold.

**Table 1:** Chemical Potencies for Envelope Formation and LDH Release^a^

compound	envelopes EC50 mg/mL (mM)	LDH release EC50 mg/mL (mM)
3-methoxycatechol	0.1 (0.07)	1 (0.07)
catechol	0.6 (0.5)	nd
pyrogallol	1 (0.8)	2 (1.6)
menadione	1 (0.6)	nd
syringol	1 (0.6)	1 (0.6)
2(5H)-furanone	6 (7)	nd
furfural	10 (10)	>50 (>52)
cyclohexanone	16 (16)	>96 (>98)
1,3-cyclohexanedione*	20 (18)	>32 (>29)
2-OH-3-methylcyclopenten-2-en-1-one	>48 (>43)	>64 (>57)
camphor	>48 (>32)	nd
maltol	>56 (>44)	3 (2.4)
m-cresol	>100 (>92)	nd
guaiacol	>100 (>81)	nd

a EC50 values given in mg/mL (and mM) were estimated for envelope formation and LDH release from concentration dependence measurements, as described in the 2 section. Envelope formation and LDH release were not observed when the EC50 value is given as greater than the highest concentration measured. nd, not determined.

A plausible mechanism for the promotion of envelope formation could involve the permeabilization of the cell membrane.^41^ Such a phenomenon, similar to the known action of ionophores,^27^ could raise the cytoplasmic calcium levels high enough to activate protein cross-linking by keratinocyte TGM1. To test the possibility that the chemicals inducing envelope formation also induced cell permeability, the release of LDH into the culture medium was measured in treated cultures. As shown in Table 1, certain chemicals induced the release of LDH and also envelope formation with similar EC50s. However, exceptions were evident. Several chemicals appeared to induce envelope formation without permeabilizing the cell membrane detectable in this way, and one (maltol) induced permeabilization but not envelope formation. These results did not rule out the possible alteration of internal calcium levels without damage to the membrane large enough for the egress of protein, but they showed that such damage detectable in this way was neither necessary nor sufficient for envelope formation.

### 3.2 Role of Transglutaminase

The possibility that the treatment of the cultures with furfural activated transglutaminase (TGM1) was explored. TGM1 protein is released from membrane anchorage by mild trypsinization of particulate extracts^42^ or by endogenous proteolysis^43^ and reportedly is a proenzyme that becomes activated by such proteolysis.^44^ To find evidence that the chemical treatment of the cultures affected the transglutaminase biochemical properties, its molecular weight and membrane anchorage were examined. As seen in Figure S1, its mobility was not altered by treatment with either furfural or syringol at concentrations promoting substantial or nearly maximal envelope formation (panels A and B). Nor was its degree of membrane anchorage altered by treatment with these chemicals (panel C).

**Figure 3:**
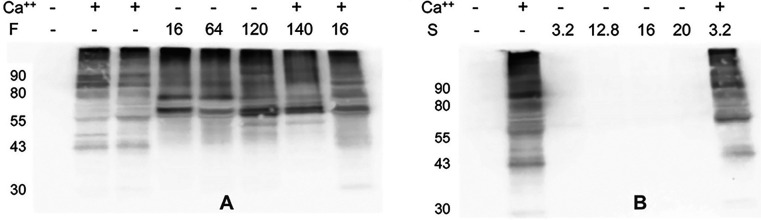
Incorporation of BPA into cellular proteins, as detected by immunoblotting. Cell extracts were incubated with the amounts (mg/mL) of either furfural (F, panel A) or syringol (S, panel B) indicated, submitted to immunoblotting with a BPA antibody. The presence (+) or absence (−) of the added calcium ion, as shown, shows the activity of endogenous TGM1 (absent in the absence of calcium).

The findings presented above point to alternative mechanisms of envelope formation in cells treated with certain smoke components. To investigate the hypotheses that some chemicals, without permeabilizing the cells, could either directly activate TGM1 or exhibit cross-linking activity themselves in the absence of TGM1, cell extracts were examined. Particulate material from keratinocyte extracts, containing membrane-bound TGM1, incorporates primary aliphatic amines into acceptor sites (glutamine residues) in certain proteins in the presence of calcium ion.^42^ This activity can be detected by immunoblotting using BPA as the substrate and an antibody that recognizes it.^29,45^ As seen in Figure 3, HEP particulates demonstrated such activity as expected in the presence but not the absence of the calcium ion. Remarkably, in the presence of the smoke constituent furfural at concentrations giving maximal envelope formation in intact cells, the incorporation of BPA was evident in the absence of calcium (Figure 3A). By contrast, treatment with syringol did not elicit this activity at concentrations giving maximal envelope formation in intact cells (Figure 3B).

This cross-linking phenomenon was further investigated by (a) inactivating transglutaminase by treating cultures with 20 mM iodoacetamide before harvesting and (b) performing the assay in parallel in human embryonic kidney 293 cells (HEK293), which express ∼1000-fold less TGM1 compared to keratinocytes.^29^ BPA incorporation was observed in cell extracts treated with furfural or 2(5H)-furanone in iodoacetamide-treated HEP as well as HEK293 extracts (Table 2). Other smoke components, including cyclohexanone, 1,3-cyclohexanedione, 1,4-cyclohexanedione, and syringol, did not promote BPA incorporation in the absence of transglutaminase activity. Thus, some smoke components appeared to induce chemical cross-linking of the proteins independent of transglutaminase-mediated isopeptide cross-linking.

**Table 2:** Biotin Pentylamine Incorporation in Human Epidermal Keratinocyte (HEP) and Human Embryonic Kidney (HEK293) Cell Extracts Treated with Individual Smoke Chemicals at Concentrations Effective in Envelope Formation^a^

**chemical**	**concentration**	**HEP**		**HEK**	
iodoacetamide	20 mM	N	Y	N	Y
calcium	5 mM	Y	N	N	N
1,4-cyclohexanedione	10 mg/mL	–	–	–	–
cyclohexanone	25 mg/mL	–	–	–	–
2(5H)-furanone	6 mg/mL	+	+	+	+
furfural	10 mg/mL	++	++	+	+
menadione	2 mg/mL	+	–	–	+
syringol	4 mg/mL	–	–	–	–

a Extracts were incubated in the presence (Y) or absence (N) of calcium chloride as indicated. Some cultures as indicated were treated (Y) or not (N) with 20 mM iodoacetamide for an hour before harvest to inactivate the TGM1 enzyme.^20^ Relative strengths of immunoreactivity are indicated by + (positive), + + (more positive), and – (negative). Representative images of blots are shown in Figure S2.

### 3.3 Envelope Protein Profiling

**Figure 4:**
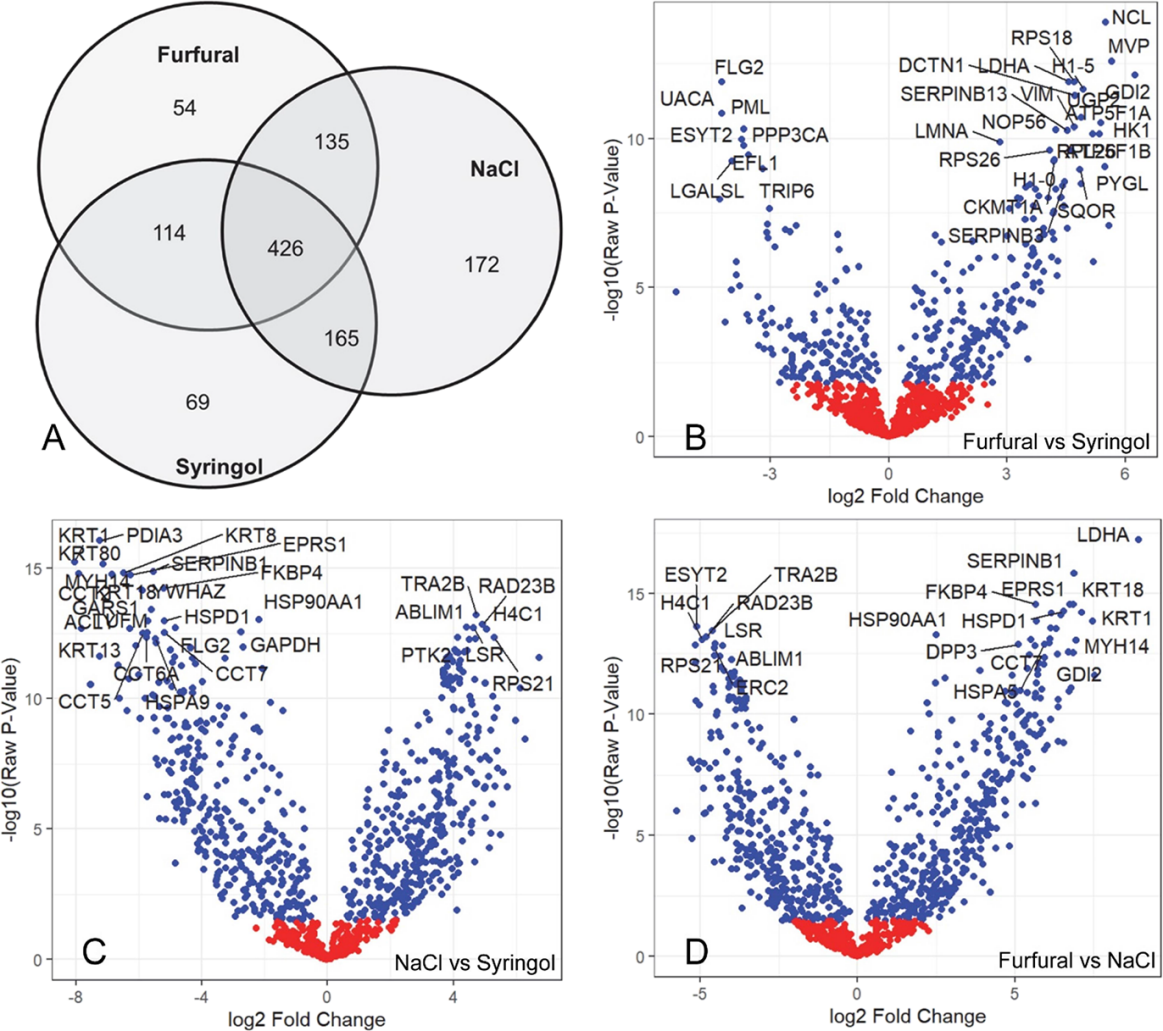
Differences in protein incorporation into envelopes elicited by the treatment with NaCl, syringol, or furfural. (A) Venn diagram shows the numbers of proteins that differed in amount in two-way comparisons of envelope profiles from the different treatments. Volcano plots show results of two-way comparisons of envelope protein profiles from these cultures. The *x*-axis shows the log2 ratio for the protein levels of furfural/syringol treatments (B), NaCl/syringol treatments (C), and furfural/NaCl treatments (D), and the *y*-axis shows −log10 of the unadjusted p-values. Protein levels that differ significantly at adjusted *p* < 0.05 are shown in blue, and the remaining proteins are shown in red.

To find whether the chemical treatment perturbed the incorporation of proteins into envelopes, protein profiling of these structures was performed with a focus on samples treated with NaCl, syringol, or furfural at concentrations just sufficient to give maximal envelope formation. Trypsin digestion, mass spectrometric analysis, and database searching identified 1162 proteins collectively in the envelope and soluble fractions of these samples. The various protein levels were distinctly different in the three groups of envelope samples, as seen in a multidimensional scaling analysis (proteomic statistical analysis, Supplementary file). When subjected to pairwise comparisons among the different treatments (Figure 4A), nearly half of the total envelope proteins were unchanged in their degree of incorporation, but the remaining proteins were differentially incorporated. (The full listing of proteins submitted for statistical analysis is shown in Table S1.) Volcano plots show results of two-way comparisons of envelope samples from cultures treated with furfural, syringol, or NaCl. Comparisons of the proteins labeled in the figure at the extremes of p value and maximal difference in expression level (fold change) show little overlap, consistent with the strikingly different profiles indicated in panel 4A. Figure S3 permits the visualization of the fold differences in the relative incorporation of individual proteins into envelopes. Each figure highlights the dramatic differences in the levels of incorporation of numerous proteins in the envelopes among the three treatment groups.

**Figure 5:**
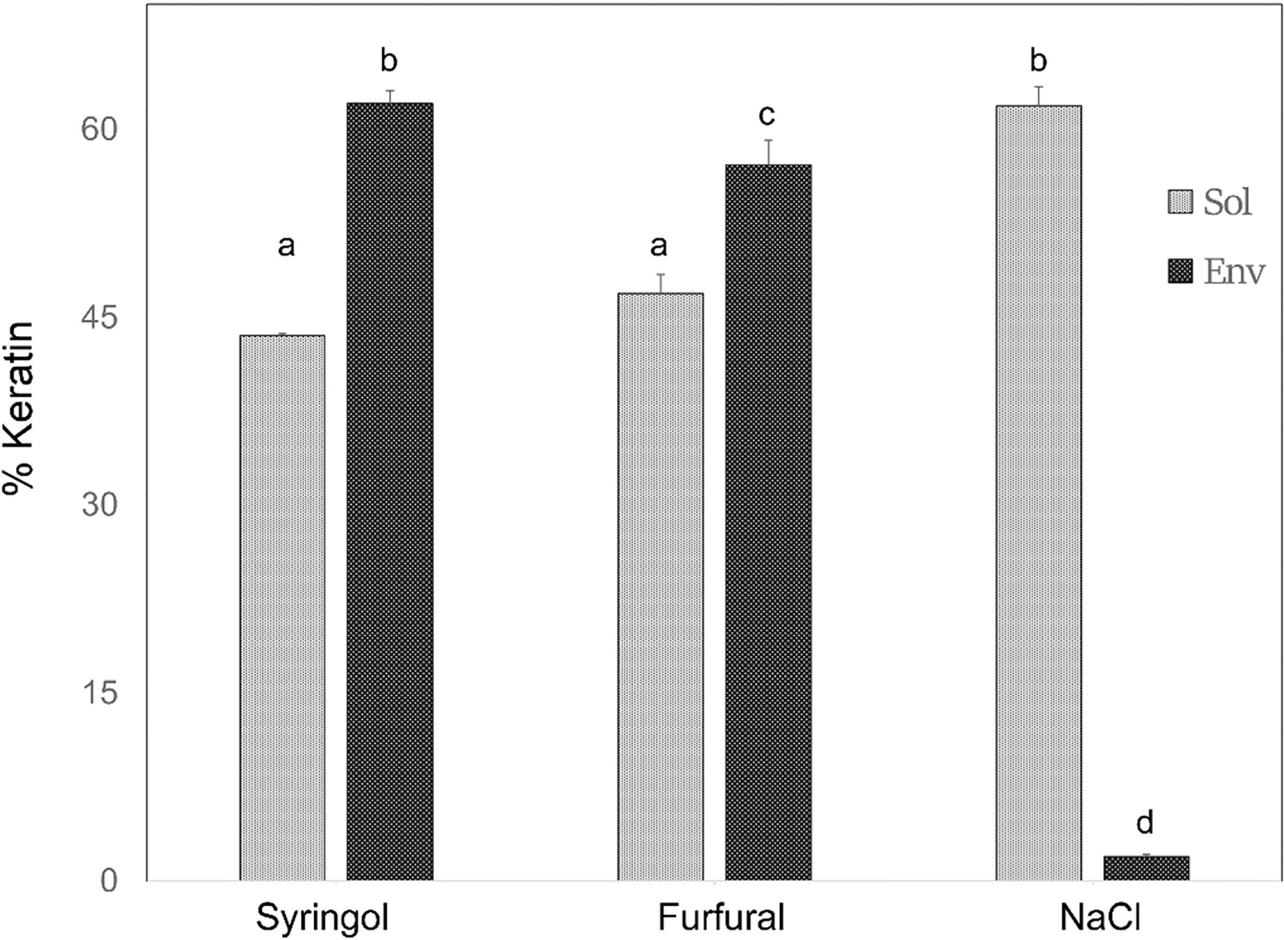
Dependence of keratin protein incorporation into envelopes on chemical treatment revealed by label-free quantitation. As shown, the % of envelope proteins (Env) that consist of keratins is low (2%) for envelopes stimulated by NaCl treatment but much higher for cultures treated with syringol or furfural. The % of keratins in the SDS/DTT soluble cell protein is similar in each case but slightly lower in cultures treated with the air pollution chemicals due to their high incorporation in envelopes. Bars with different labels (a–d) were statistically different in % keratin (*p* < 10^–5^).

Estimates were made by label-free quantitation of the relative amounts of each protein in the envelopes. Based on the profiles of envelope fractions from human corneocytes of epidermis, nail plate, and hair shaft,^21^ the envelope profile provides a sampling of the protein content of the cell of origin. Comparing the levels of given proteins in the envelope and soluble fractions (Table S3) showed considerable variation in the degree of enrichment in envelopes. Those proteins most obviously incorporated similar to their amounts in the soluble fraction were keratins in samples treated with syringol and furfural. Although the soluble protein fraction from each treatment was similar in profile in the present work, the envelope protein profile of cultures treated with NaCl differed dramatically from those of cultures treated with syringol or furfural. Corneocyte envelopes from epidermal callus, similar to those of the hair shaft and nail plate, have a high content (≈70%) of keratin.^21^ Envelopes induced in culture using syringol or furfural approached such a high content of keratin (≈50%), but those permeabilized with 0.8 M NaCl (2%) were much lower (Figure 5). These findings resemble the relatively high keratin content for envelope formation induced by liquid smoke extract (50%) but much lower (2%) for cells permeabilized with the reactive oxygen generating chemical DMNQ or the ionophore X537A.^20^

### 3.4 Gene Expression

**Figure 6:**
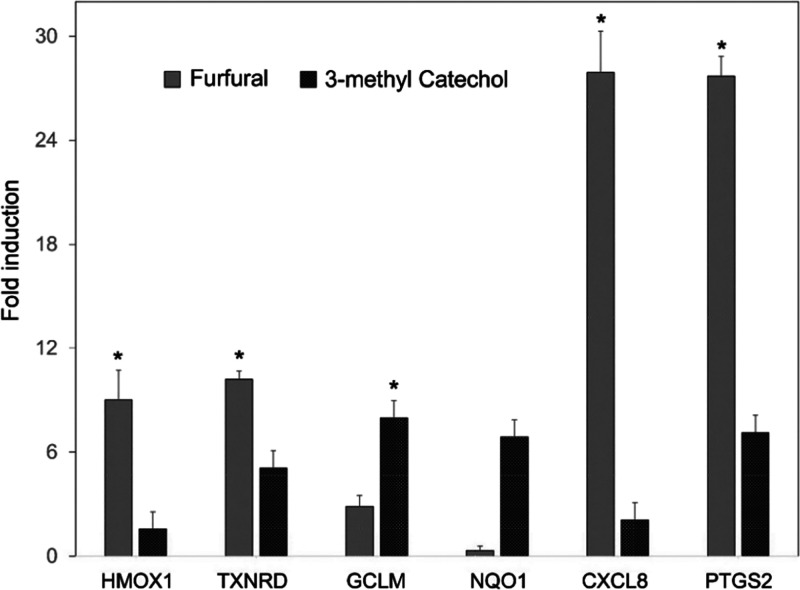
Stimulation of pro-oxidant and proinflammatory transcription by furfural and 3-methycatechol. Treating cultures with chemicals at 10% of their EC50 values for envelope formation induced HMOX1, TXNRD, CLCl8, and PTGS2 significantly above background levels (*, *p* < 0.05). 3-Methyl catechol significantly induced GCLM at that low concentration.

Expression of genes related to oxidative stress (GCLM, HMOX1, NQO1, and TXNRD) and inflammatory responses (CXCL8 and PTGS2) was measured using TaqMan assays with qPCR. Since treatment at concentrations near the EC50 value for stimulating envelope formation (a lethal event) can lead to reduced mRNA yield,^29^ transcriptional effects were measured at concentrations considerably lower, 10% of the EC50 value. Among the chemicals assayed, furfural gave the most effective response at this concentration after incubation for 1 day, with induction close to or above 10-fold for HMOX1, TXNRD, CXCL8, and PTGS2 above background (Figure 6). Induction of GCLM was lower (≈threefold, not statistically significant), and NQO1 induction was not clearly different from the baseline. The structurally similar furanone gave considerably less induction than furfural (not significantly elevated), and syringol produced little or no induction of these genes (neither shown). By contrast, 3-methoxycatechol at 10% of its EC50 gave significantly elevated values only for GCLM, but at its EC50 produced substantially higher induction than furfural for all the genes assayed except NQO1, and responses for HMOX1, GCLM, and PTGS2 were significantly above the background (Figure S4).

**Figure 7:**
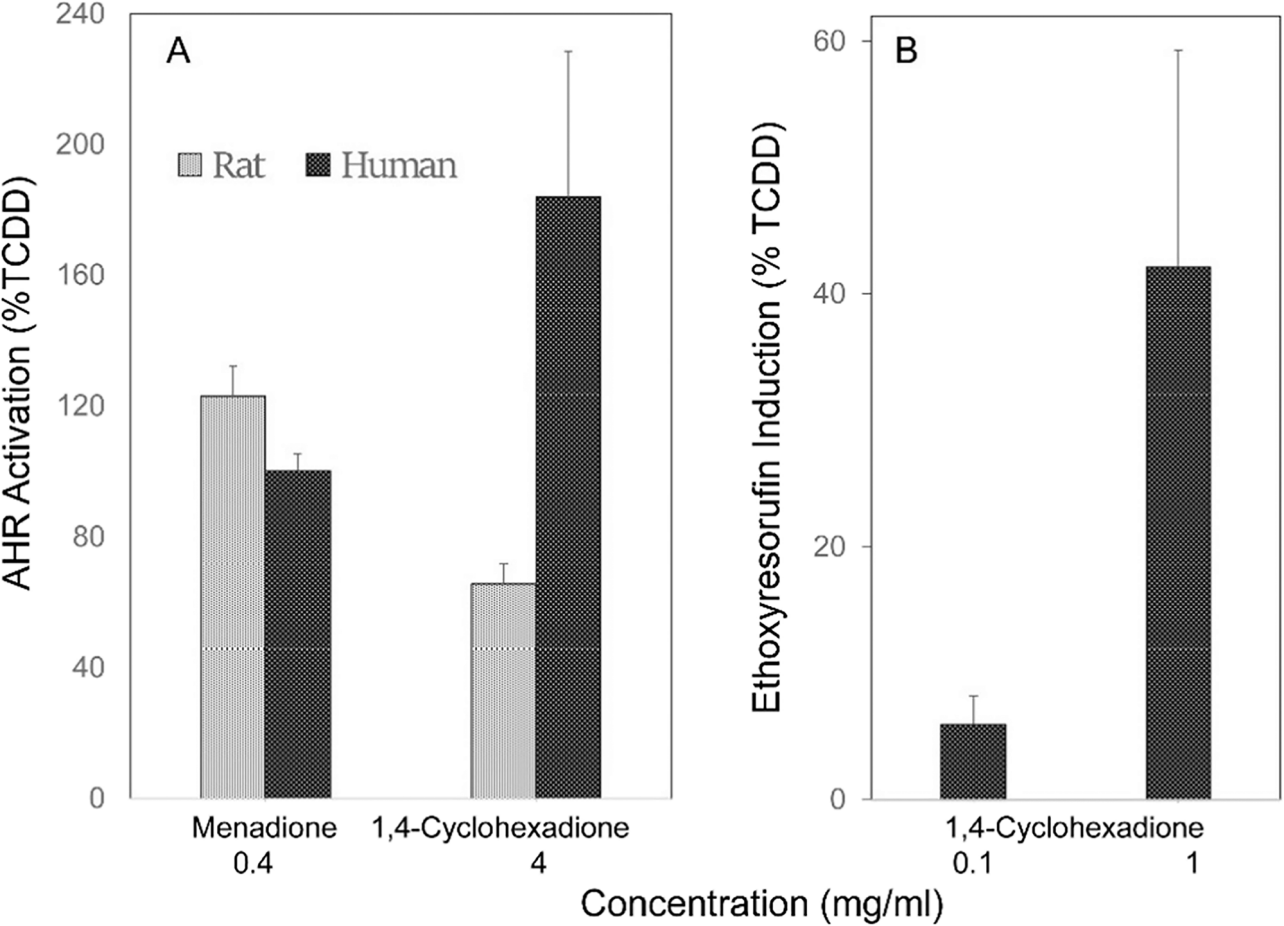
Activation of the aryl hydrocarbon receptor (A) and 7-ethoxyresorufin O-deethylase expression (B) by 1,4-cyclohexanedione. Cyclohexanedione and menadione were active in rat and human hepatoma lines carrying a dioxin response element (A), but only cyclohexanedione was active in inducing CYP1A1 in human keratinocytes (B). (A) Values of each bar were significantly different from background (0), *p* < 0.02. (B) Value for 1 mg/mL was significantly different from 0.1 mg/mL (*p* < 0.002), which was not significantly different from background (0 mg/mL).

The paucity of polycyclic aromatic hydrocarbons in liquid smoke raised the question of which chemicals were responsible for inducing CYP1A1 in the keratinocytes. The AHR is known to be activated by a profusion of environmental chemicals, generally hydrophobic and of low potency.^46^ Woodsmoke contains a plethora of complex products, many derived from cellulose (furfural-related) and lignin (guaiacol, syringol, and dimers).^9^ Screening of specific compounds for AHR activation using human and rat hepatoma lines with a luciferase reporter^29^ showed little or no activity toward numerous compounds (including acetosyringone, camphor, catechol, m-cresol, furfural, 2(5H)-furanone, maltol, 3-methoxycatechol, 5-methylfurfural, syringol, and vanillin). However, surprisingly, pyrogallol, menadione, and diones of cyclohexane tested positive. When these were tested in human epidermal cell cultures for the induction of ethoxyresorufin O-deethylase activity, however, only cyclohexanediones produced a substantial response (Figure 7). Menadione and pyrogallol, which were positive in the AHR activation in hepatoma cells, showed little or no activity in the human keratinocytes.

## 4 Discussion

As an initial step to identify woodsmoke constituents with biological activities, the present work focused on those responses in keratinocytes observed with liquid smoke extract.^29^ The results indicate that individual chemicals can be identified that contribute to the biological effects of woodsmoke. Continued efforts in this direction may yield surprising results. For example, the unexpected activity of 1,4-cyclohexanedione in both hepatoma cells used for high-throughput screening and in human keratinocytes adds to the possible ways such chemicals could interact with and activate the AHR. In contrast, activation by pyrogallol and menadione in the hepatoma lines likely reflects their induction of reactive oxygen species generating tryptophan pathway catabolites that are receptor ligands,^47,48^ a propensity that could differ among cell types with different levels of antioxidant defense.

The observation long ago, that permeabilizing keratinocytes with dilute nonionic detergent, high salt concentration, or ionophores such as X537A induced envelope formation by activating keratinocyte transglutaminase,^27^ appears superficially consistent with the action of several compounds tested. Thus, syringol and pyrogallol induced envelopes and the release of LDH from the cells at 1–2 mg/mL. However, the potencies of other compounds differed greatly in the two actions. The ability of 3-methoxycatechol to induce oxidative stress could rationalize its much greater potency in envelope formation, but maltol induced LDH release at 3 mg/mL without inducing envelope formation. The latter observation indicates that LDH release itself is not diagnostic of the calcium mobilization inside the cell that is ordinarily required for TGM1 activation.

Volatile organics in woodsmoke and other air pollution are well-known to undergo continuing oxidation and related reactions downwind of emission, where oxygenated nonaromatics dominate.^49^ Analogous alteration of the chemicals used to treat the cells likely occurs during incubation for a day at 37 °C in the present work. Oxidative stress as a consequence of pollutant exposure appears to be responsible for adverse health effects, but reactions of carbonyls are capable of exacerbating cell responses. This phenomenon is evident in the skin from the studies of cosmetic and fragrance chemicals through Schiff base formation by protein adducts^50^ and plausibly interacting with KEAP1 thiols to activate Nrf2 signaling.^51^ Such reactions likely rationalize how smoke chemicals can contribute to eliciting inflammatory responses. In view of the limitations of peptide reaction assays,^52^ however, identifying the protein adducts of complex mixtures will be a formidable task. Nevertheless, elucidating reactions of single chemicals with proteins in cells could help interpret findings of transcriptomic analyses of exposure groupings.^53^

Efforts are underway to identify reactive chemicals in air pollution responsible for forming adducts of hemoglobin and serum albumin in human blood samples.^54^ Such measures could be useful in biomonitoring exposure to pollutants that form macromolecular adducts that are responsible for adverse effects. A promising pilot study in smokers, using a sensitive cysteine-containing albumin peptide as a marker, revealed an increase in albumin adducts compared to nonsmokers,^55^ but finding a correlation of such adducts to outcomes from short-term air pollution exposures has proven challenging.^56^ Exposure levels in the present work for single chemicals are much higher than those in real-world scenarios, particularly for envelope formation. However, finding that total protein incorporation into envelopes is greatly increased provides strong support for a chemically induced increase in protein cross-linking.

We speculate that the continuing degradation of smoke chemicals evolves reactive oxygen and carbonyl compounds, both of which participate in forming covalently linked macromolecular structures. This can be readily visualized in the case of catechol derivatives, the most potent chemicals tested, which autooxidize to give reactive oxygen and quinones. It has long been known that quinones are involved in protein polymerization in vivo^57^ and that reactive oxygen can yield cross-linking at tyrosine residues in heme proteins.^58,59^ In addition, protein carbonylation likely occurs, providing other cross-linking opportunities.^60^ By analogy with the products of protein browning (Maillard) reactions with reducing sugars and even dihydroxyacetone,^61^ complications may arise from a combination of Schiff’s base formation, aldol condensation, and Michael addition of smoke chemicals and their oxidized products with diverse protein nucleophiles. Such reactions are likely to occur even at much lower exposure levels than those used presently that frequently gave products with a light tan color. Identifying some mechanisms by which the cross-linking occurs and susceptible sites in proteins promises to elucidate important mechanisms responsible for deleterious effects of air pollution and may provide sensitive additional pathways to monitor individual exposures.

## Supporting Information Available

TGM1 shows little if any change in molecular weight or membrane binding by immunoblotting upon treatment with smoke chemicals; representative images of biotin pentylamine incorporation in human epidermal (HEP) and embryonic kidney (HEK-293) cell extracts treated with individual smoke components without (IA-) or with (IA+) pretreatment with iodoacetamide; differences in protein incorporation into envelopes by NaCl, syringol, and furfural; stimulation of oxidative stress-responsive gene transcription by 3-methoxycatechol; induction of HMOX1, GCLM, and PTGS2 was significantly above background by ANOVA (*p* < 0.05); proteomic statistical analysis; analysis of protein profiles by multidimensional scaling plot of sample results and R script for the analysis (PDF)
Exclusive spectral counts of the envelope (E) and solubilized (S) protein fractions from treated cultures; label-free quantitation of solubilized (S) and envelope (E) protein amounts; summed areas of the top three proteotypic peptides for each sample, followed by values normalized to a total of 100 for each sample; relative protein amounts in the envelope and soluble fractions; proteins differing in relative amounts in soluble versus envelope fractions from cultures treated with NaCl (S4), syringol (S5), and furfural (S6); proteins differing in relative amounts in envelopes from cultures treated with NaCl and syringol (S7), NaCl and furfural (S8), and syringol and furfural (S9); and proteins differing in relative amounts in the soluble fraction from cultures treated with NaCl and syringol (S10), NaCl and furfural (S11), and syringol and furfural (S12) (XLSX)

## ABBREVIATIONS

AHR: aryl hydrocarbon receptor
BPA: biotin pentylamine;
CXCL8: interleukin 8
CYP: cytochrome P450
DTT: dithioerythritol (used interchangeably with dithioerythritol)
ERC50: effective concentration giving 50% maximal response
EROD: ethoxyresorufin O-deethylase
GAPDH: glyceraldehyde phosphate dehydrogenase
GCLM: glutamate-cysteine ligase modifier subunit
GUSB: beta-glucuronidase
HEP: human epidermal keratinocytes
HMOX1: heme oxygenase 1
NQO1: NADPH quinone acceptor oxidoreductase 1
PBS: phosphate-buffered saline
qPCR: quantitative (real time) PCR
SDS: sodium dodecyl sulfate
TCDD: 2,3,7,8-tetrachlorodibenzo-p-dioxin
TGM1: transglutaminase-1
PTGS2 (COX2): prostaglandin-endoperoxide synthase 2
TXNRD: thioredoxin reductase
