# Supplementary material for: Human Keratinocyte Responses to Woodsmoke Chemicals
Source: Chem Res Toxicol. 2024 Apr 10;37(5):675–84. doi: 10.1021/acs.chemrestox.3c00353 (PMC11110105; doi:10.1021/acs.chemrestox.3c00353)
Supplement: Supplementary file 1 — tx3c00353_si_001.pdf [file tx3c00353_si_001.pdf]

Supporting Information

Human Keratinocyte Responses to Woodsmoke Chemicals

*Noreen Karim†¶, Yatian Yang†‖, Michelle Salemi‡, Brett S. Phinney‡, Blythe P. Durbin-Johnson§, David M. Rocke§, Robert H. Rice†**

*Corresponding author: Email rhrice@ucdavis.edu

†Department of Environmental Toxicology, University of California, Davis, CA USA

‡Proteomics Core Facility, University of California, Davis, CA USA

§Division of Biostatistics, Department of Public Health Sciences, Clinical and Translational Science Center Biostatistics Core, University of California, Davis CA USA

Contents — Page

Figure S1. TGM1 shows little if any change in molecular weight or membrane binding by immunoblotting upon treatment with smoke chemicals. — S2

Figure S2. Representative images of biotin pentylamine incorporation in human epidermal (HEP) and embryonic kidney (HEK-293) cell extracts treated with individual smoke components without (IA-) or with (IA+) pretreatment with iodoacetamide. — S3

Figure S3. Differences in protein incorporation into envelopes by NaCl, syringol and furfural. — S4

Figure S4. Stimulation of oxidative stress-responsive gene transcription by 3-methoxycatechol. — S5

Proteomic Statistical Analysis. Analysis of protein profiles by multidimensional scaling plot of sample results and R script for the analysis. — S6 -7

Table S1. Exclusive spectral counts of the envelope (E) and solubilized (S) protein fractions from treated cultures. — Excel

Table S2. Label fee quantitation of solubilized (S) and envelope (E) protein amounts. — Excel

Table S3. Relative protein amounts in envelope and soluble fractions. — Excel

Tables S4-S12. Proteins differing in relative amount in soluble versus envelope fractions from cultures. — Excel

Supplementary Figures

Figure S1. TGM1 shows little if any change in molecular weight or membrane binding by immunoblotting upon treatment with smoke chemicals. (A) and (B) show little change in electrophoretic mobility using antibodies to the C-terminus (A) or an internal epitope (B). (C) shows retention of membrane binding after 24 h of treatment.

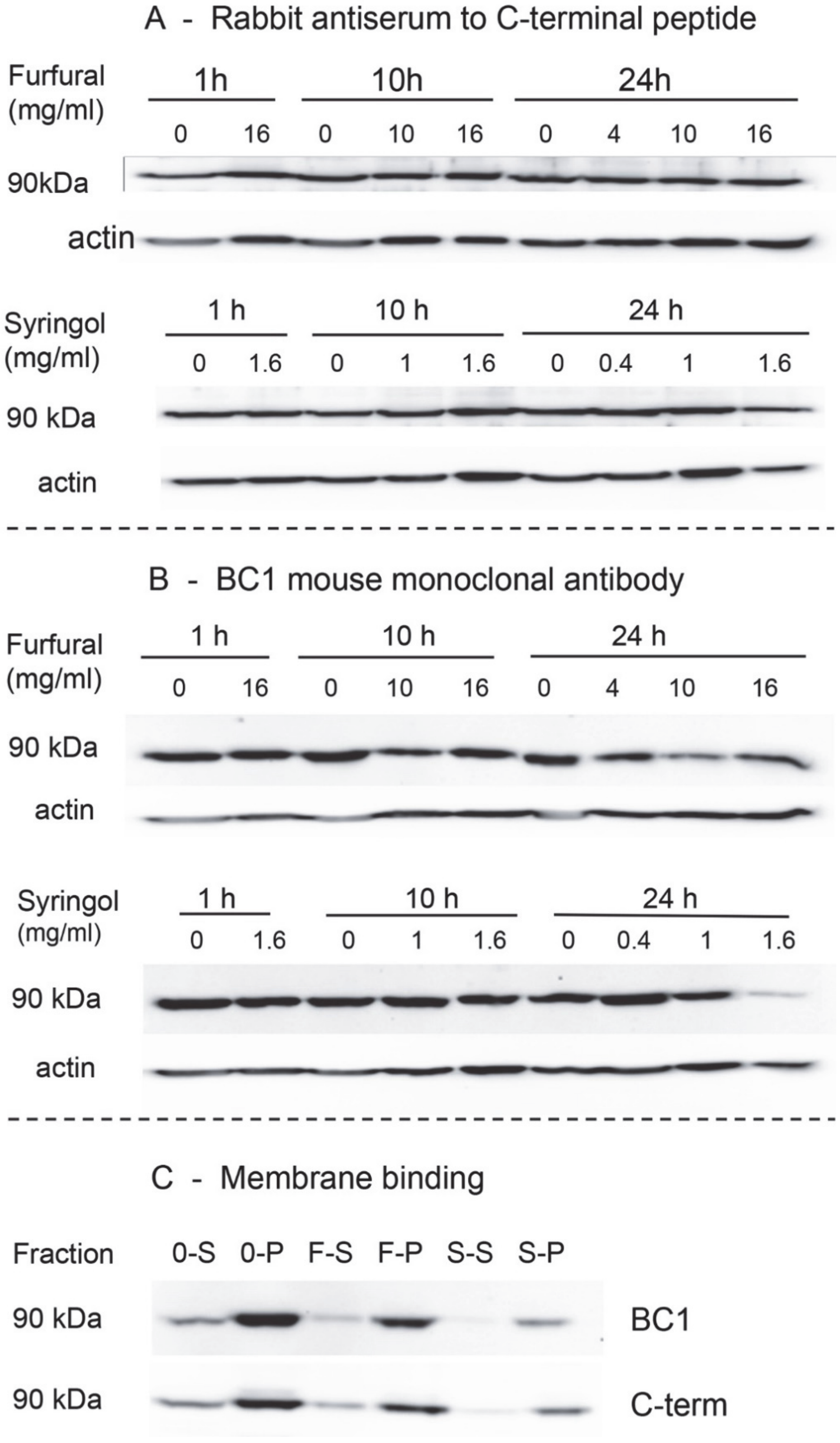

Figure S2. Representative images of biotin pentylamine incorporation in human epidermal (HEP) and embryonic kidney (HEK-293) cell extracts treated with individual smoke components without (IA-) or with (IA+) pretreatment with iodoacetamide. The degree of biotin incorporation is indicated by the darkness of the image. Additions to the extracts are indicated above the lanes: Ca++, Calcium; Fon, 2-Furanone; Fur, Furfural; Men, Menadione; Syr, Syringol; 1,4Chd, 1,4-Cyclohexanedione; Ch, Cyclohexanone; 1,3Chd, 1,3-Cyclohexanedione. For Men*, the asterisk indicates the extracts without and with iodoacetamide treatment were switched.

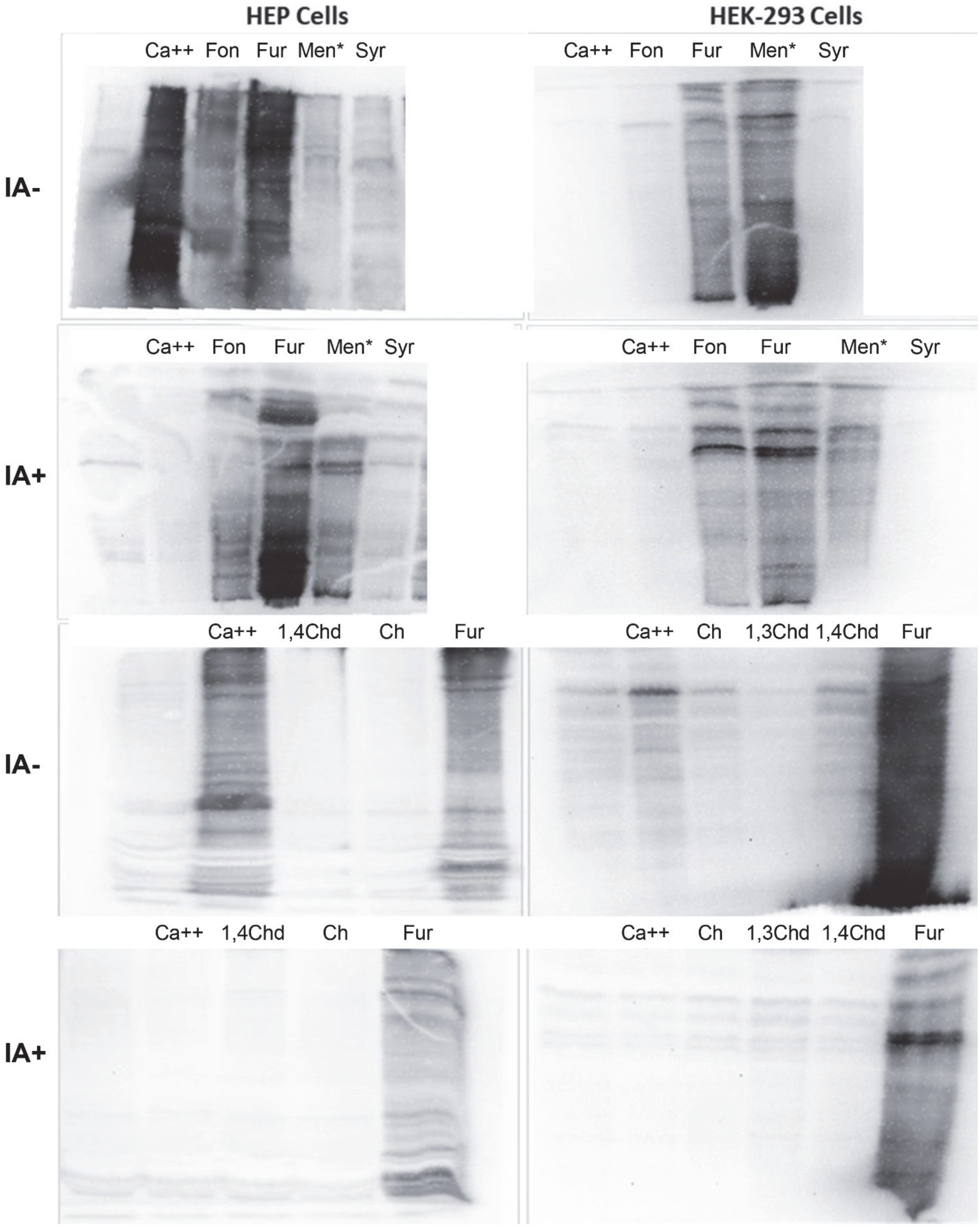

Figure S3. Differences in protein incorporation into envelopes by NaCl, syringol and furfural. For each treatment, the relative amounts of each protein incorporated into envelopes compared to solubilized proteins (Env/Sol) were calculated. These ratios are plotted on the vertical scale with increasing fraction in the envelopes on the horizontal scale. Values for NaCl (blue line), syringol (orange circles) and furfural (open circles) are shown as a scatter plot for the three treatments.

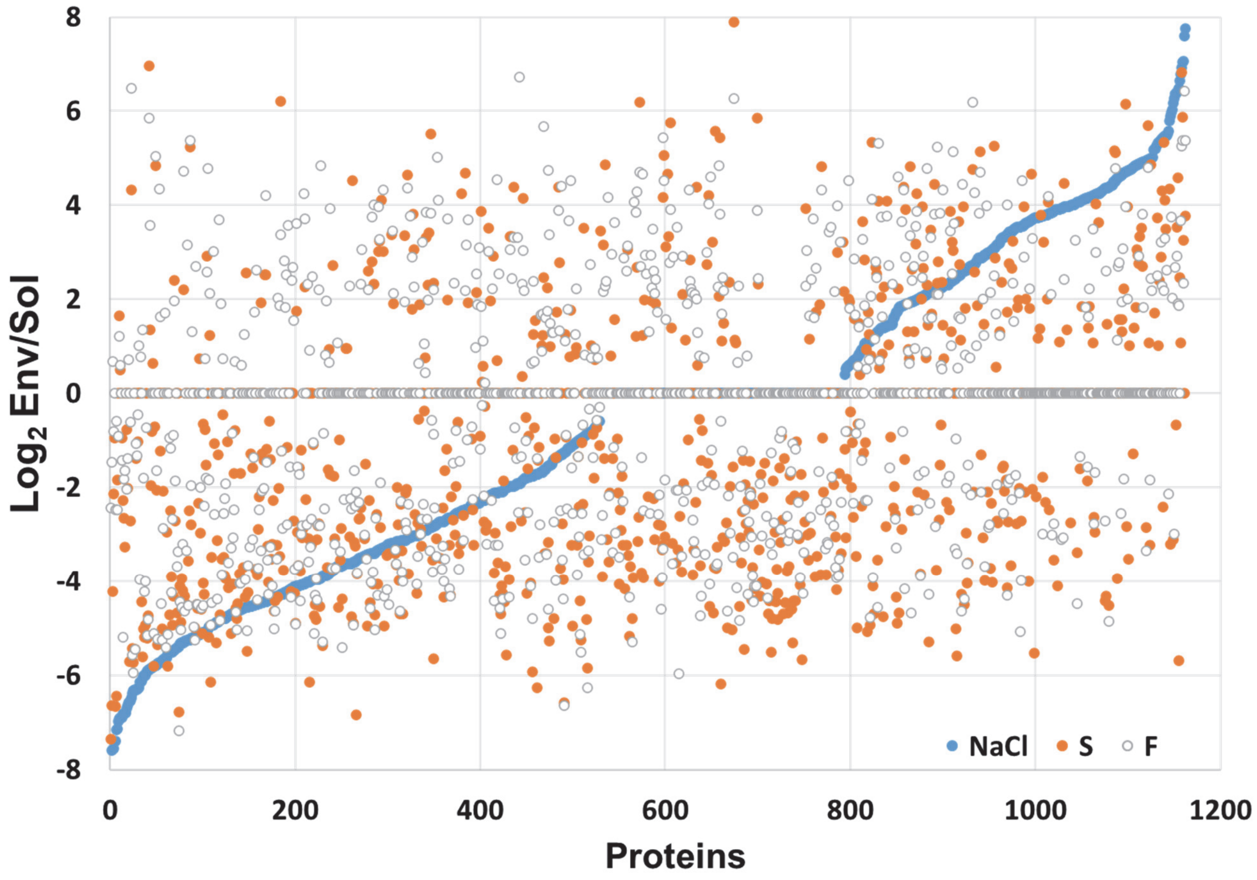

Figure S4. Stimulation of oxidative stress-responsive gene transcription by 3-methoxycatechol. Inductions of HMOX1, GCLM and PTGS2 were significantly above background by ANOVA ($p < 0.05$).

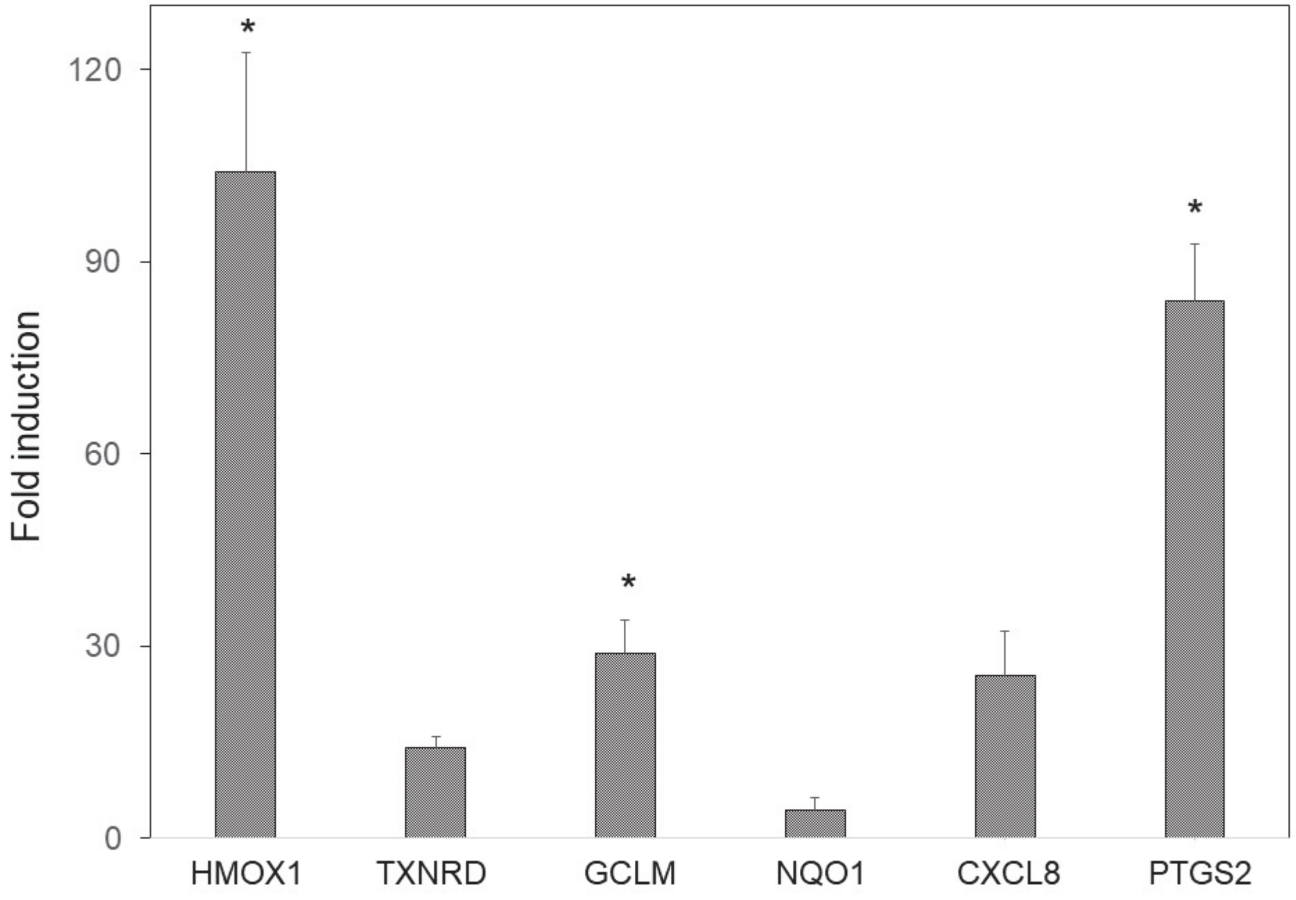

Proteomic Statistical Analysis – Multidimensional scaling plot and R script

Blythe Durbin-Johnson, Ph.D. 2020-11-13

References

1. Robinson MD, Oshlack A (2010). A scaling normalization method for differential expression analysis of RNA-seq data. Genome Biology 11, R25.

2. Ritchie, M.E., Phipson, B., Wu, D., Hu, Y., Law, C.W., Shi, W., and Smyth, G.K. (2015). limma powers differential expression analyses for RNA-sequencing and microarray studies. Nucleic Acids Research 43(7), e47.

Multidimensional scaling (MDS) plot by chemical and fraction

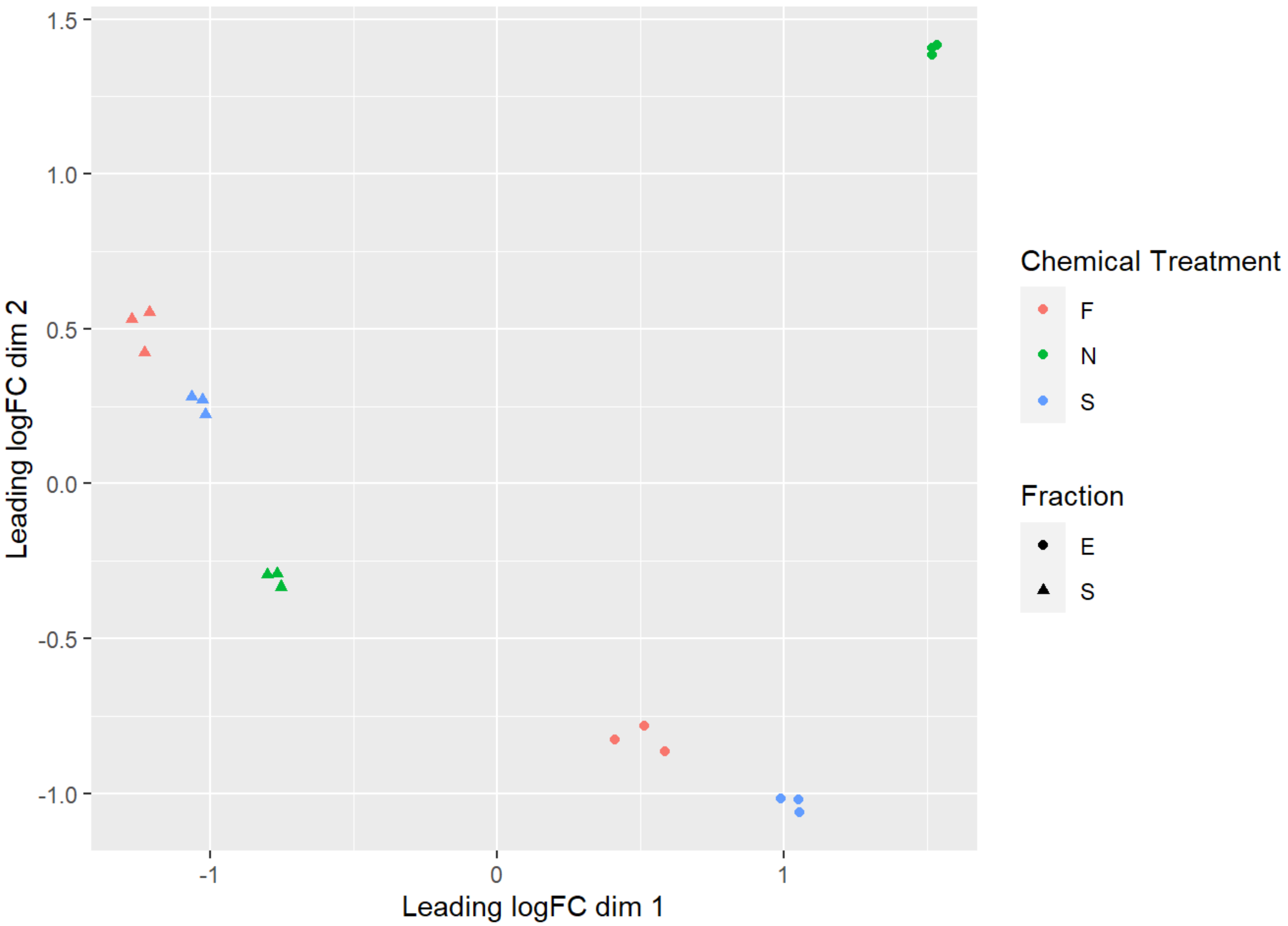

Results of two way comparisons are given in Tables S3-S11.

R session information

```
## R version 4.0.2 (2020-06-22)
## Platform: x86_64-w64-mingw32/x64 (64-bit)
## Running under: Windows 10 x64 (build 17134)
##
## Matrix products: default
##
## locale:
## [1] LC_COLLATE=English_United States.1252
## [2] LC_CTYPE=English_United States.1252
## [3] LC_MONETARY=English_United States.1252
## [4] LC_NUMERIC=C
## [5] LC_TIME=English_United States.1252
##
## attached base packages:
## [1] stats     graphics  grDevices datasets  utils     methods   base
##
## other attached packages:
## [1] kableExtra_1.3.1 knitr_1.30       emmeans_1.5.2-1  dplyr_1.0.2
## [5] ggplot2_3.3.2    edgeR_3.32.0     limma_3.46.0     readxl_1.3.1
##
## loaded via a namespace (and not attached):
##  [1] Rcpp_1.0.5        highr_0.8         plyr_1.8.6        cellranger_1.1.0
##  [5] compiler_4.0.2    pillar_1.4.6      tools_4.0.2       digest_0.6.27
##  [9] viridisLite_0.3.0 evaluate_0.14     lifecycle_0.2.0   tibble_3.0.4
## [13] gtable_0.3.0      lattice_0.20-41   pkgconfig_2.0.3   rlang_0.4.8
## [17] rstudioapi_0.13   yaml_2.2.1        mvtnorm_1.1-1     xfun_0.19
## [21] xml2_1.3.2        httr_1.4.2        withr_2.3.0       stringr_1.4.0
## [25] generics_0.1.0    vctrs_0.3.4       webshot_0.5.2     locfit_1.5-9.4
## [29] grid_4.0.2        tidyselect_1.1.0  glue_1.4.2        R6_2.5.0
## [33] rmarkdown_2.5     farver_2.0.3      purrr_0.3.4       magrittr_1.5
## [37] scales_1.1.1      htmltools_0.5.0   ellipsis_0.3.1    rvest_0.3.6
## [41] xtable_1.8-4      colorspace_2.0-0  renv_0.12.2       labeling_0.4.2
## [45] estimability_1.3  stringi_1.5.3     munsell_0.5.0     crayon_1.3.4
```
